# Supplementary material for: Pervasive isoform‐specific translational regulation via alternative transcription start sites in mammals
Source: Mol Syst Biol. 2016 Jul 18;12(7):875. doi: 10.15252/msb.20166941 (PMC4965872; doi:10.15252/msb.20166941)
Supplement: Supplementary file 1 — Expanded View Figures PDF [file MSB-12-875-s001.pdf]

Expanded View Figures

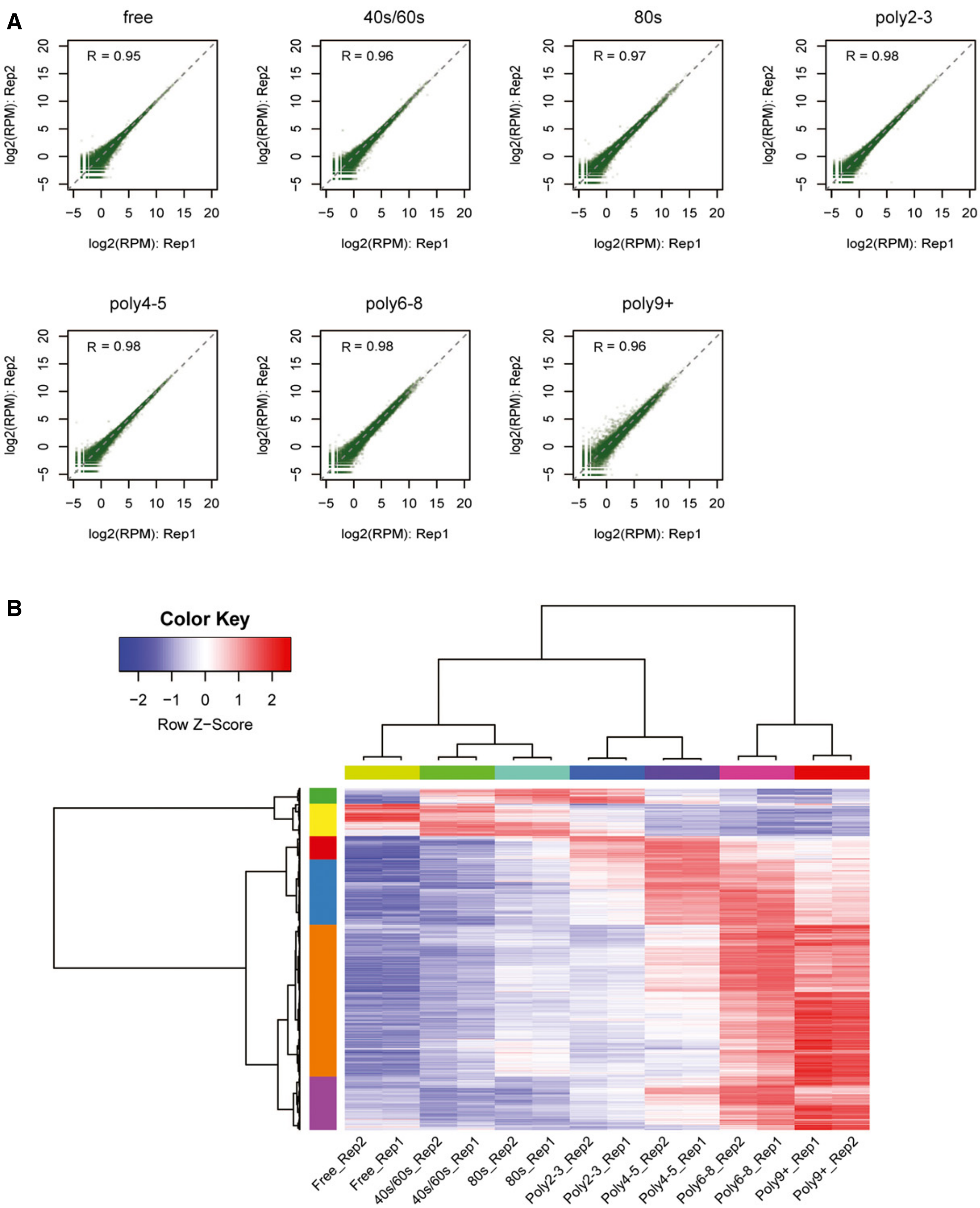

Figure EV1. TSS discovery and quantification across different gradient fractions detected in this study.

A The scatter plot comparing read counts of each TSS cluster between biological replicates for each of the seven polysome fractions.

B Hierarchical clustering of TSS isoform abundance across all fractions and replicates. Each row represented one TSS isoform, and each column represented different fractions. *Z*-scores, showing in different colors, represented the normalized isoform abundance across fractions.

© 2016 The Authors

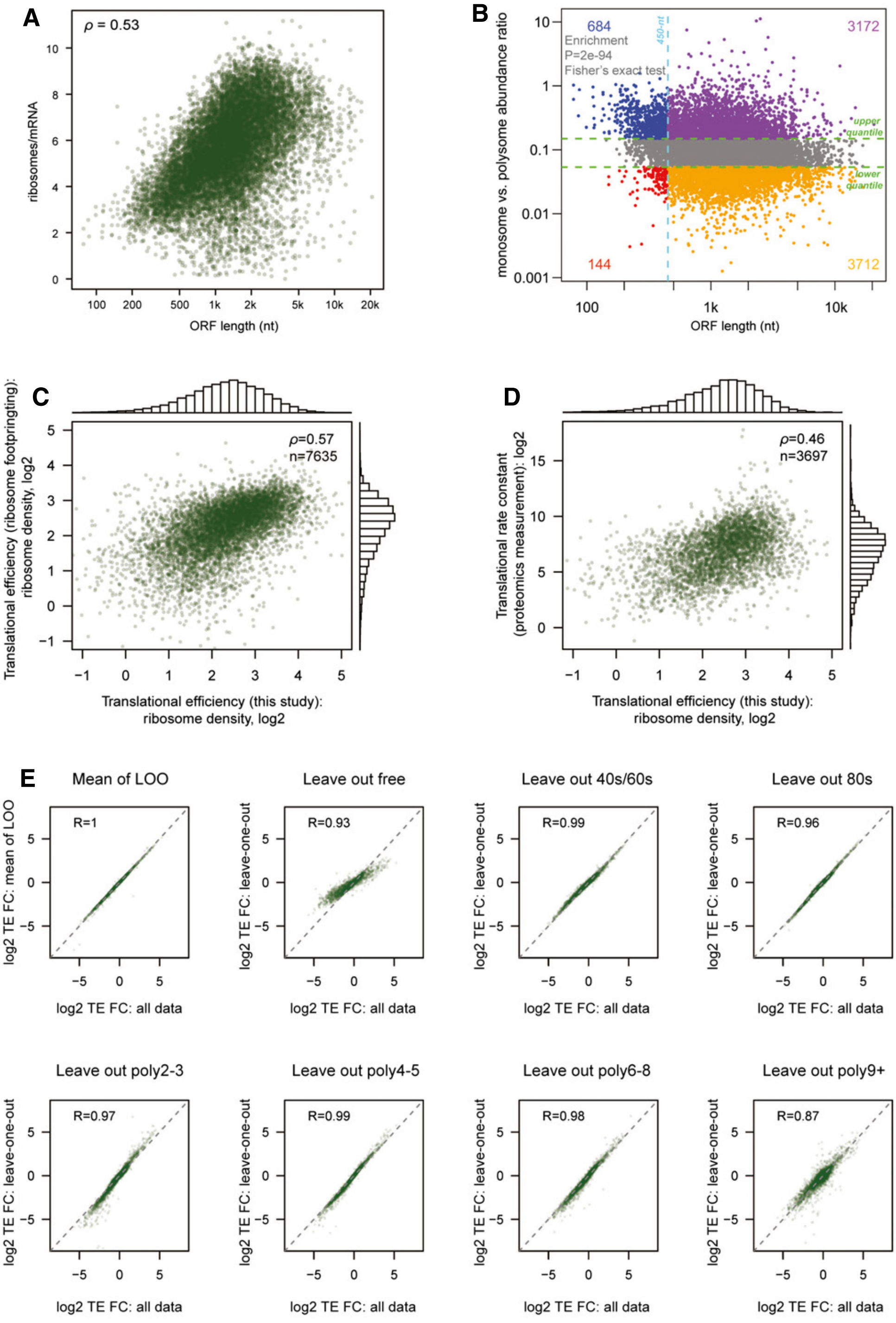

Figure EV2. Polysome profiling measured mRNA translational efficiency.

A For each TSS isoform, the number of ribosomes per mRNA was plotted against its corresponding ORF length.

B For each TSS isoform, the abundance ratio between monosome fraction and sum of polysome factions was plotted against its corresponding ORF length. Short ORFs (≤ 450 nt) were more enriched in the monosome fraction.

C TE values for each gene calculated based on published ribosome footprinting data were plotted against the TE values calculated based on polysome profiling data in this study.

D TE values for each gene calculated based on published proteomics/genomics data were plotted against the TE values calculated based on polysome profiling data in this study.

E Log2-transformed TE fold change values for each pair of alternative TSS isoforms calculated based on all seven fraction data were compared to those calculated based on data with one of the seven fractions left out.

 © 2016 The Authors

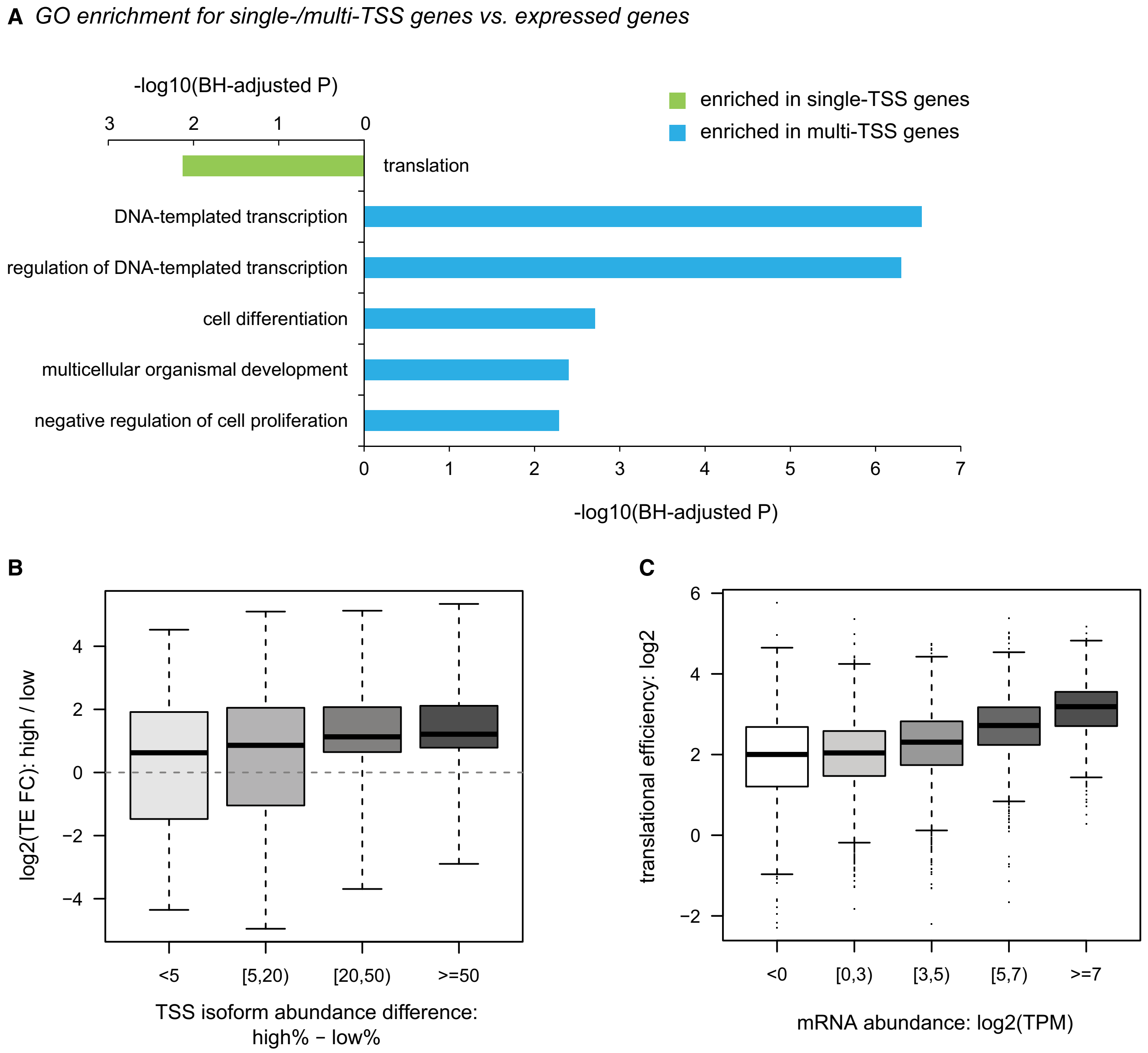

Figure EV3. GO enrichment analyses and the relationship between transcription and translation.

A GO enrichment for single/multi-TSS genes over all expressed genes.

B Boxplots showing the distribution of TE divergence between alternative TSS isoforms grouped by their abundance differences. Box edges represent quantiles, whiskers represent extreme data points.

C Boxplots showing the distribution of TE at the gene level grouped by mRNA abundance. Box edges represent quantiles, whiskers represent extreme data points no more than 1.5 times the interquartile range.

© 2016 The Authors

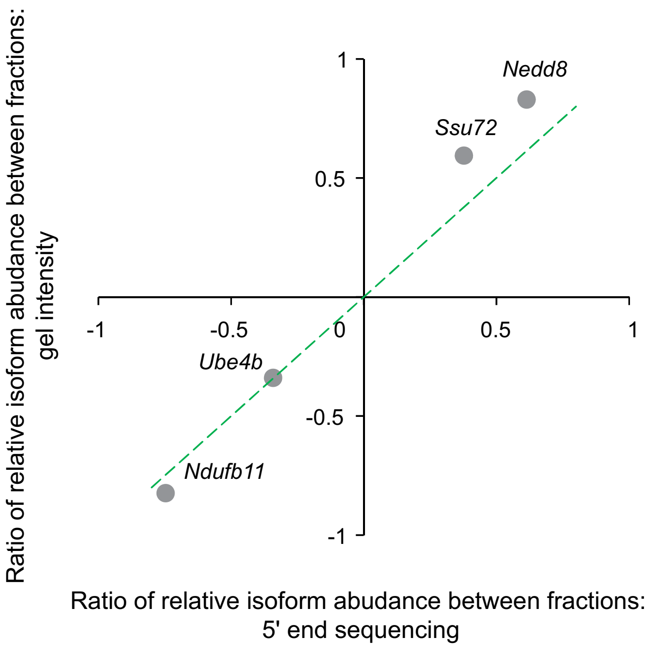

Figure EV4. Quantitative validation of TE divergence between TSS isoforms.

The ratio of relative isoform abundance between fractions was calculated according to the formula $\frac{T_{dist,poly}/T_{prox,poly}}{T_{dist,nonribo}/T_{prox,nonribo}}$, where $T_{dist,poly}$ and $T_{prox,poly}$ represented the isoform abundance of distal and proximal TSSs in the polysomal fraction, respectively; $T_{dist,nonribo}$ and $T_{prox,nonribo}$ represented the isoform abundance in the non-ribosomal fraction. The ratio determined based on agarose gel image (*y*-axis) was plotted against that estimated based on 5′ end sequencing (*x*-axis).

 © 2016 The Authors

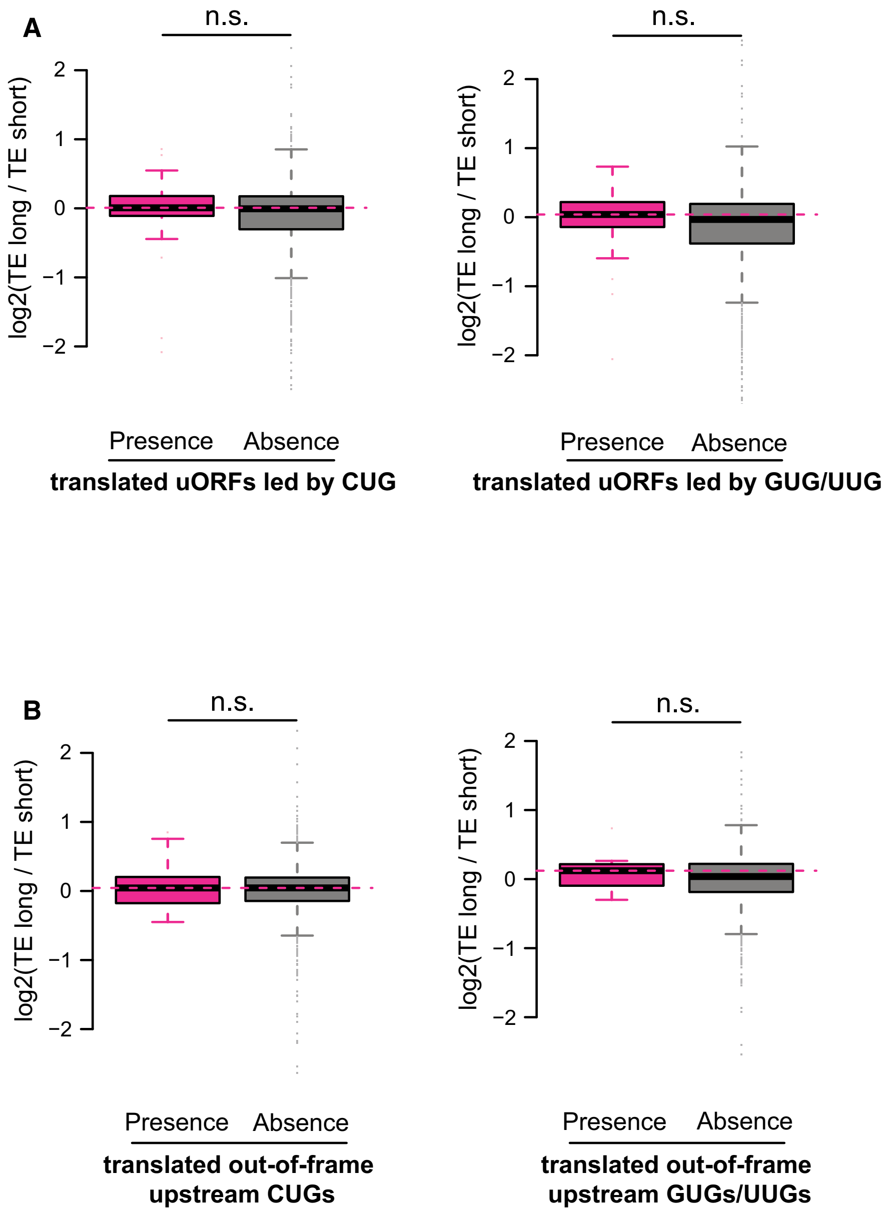

Figure EV5. Upstream translation at non-AUG start codon had no significant impact on main ORF translation.

A Similar to Fig 4F, but split non-canonical uORFs into uORFs led by CUGs and uORFs led by GUGs/UUGs.

B Similar to Fig 4G, but split out-of-frame upstream non-canonical start codons into out-of-frame upstream CUGs and out-of-frame upstream GUGs/UUGs.

Data information: Box edges represent quantiles, whiskers represent extreme data points no more than 1.5 times the interquartile range.

© 2016 The Authors

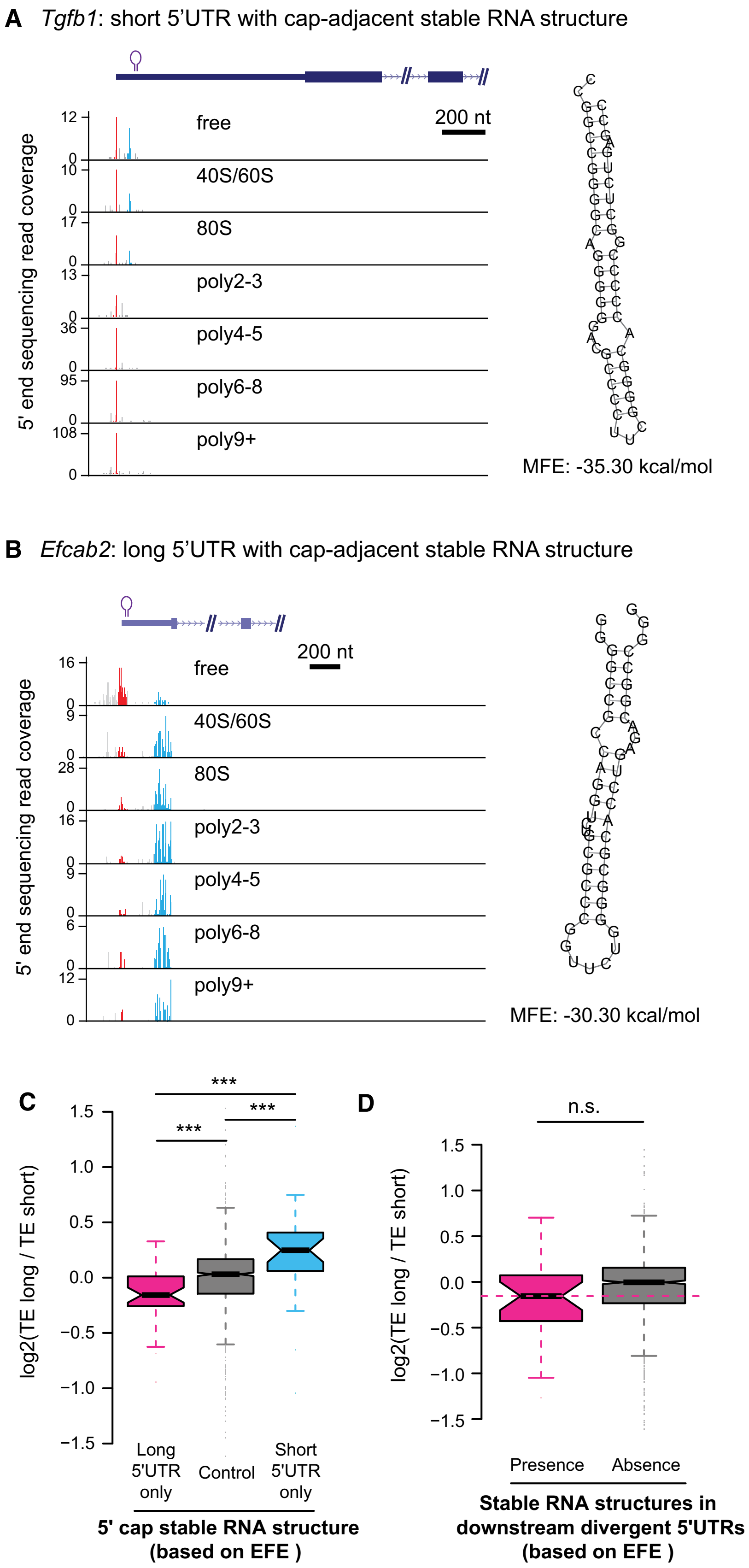

Figure EV6. Cap-adjacent stable RNA structures inhibit translation.

A, B Two examples showing that cap-adjacent stable RNA structures repressed translation. The description of the two genes can be found in Table EV3.

C, D Same as Fig 5A and B, but based on EFE to define stable RNA structures. ***$P < 0.001$; Mann–Whitney U-test. Box edges represent quantiles, whiskers represent extreme data points no more than 1.5 times the interquartile range.

© 2016 The Authors

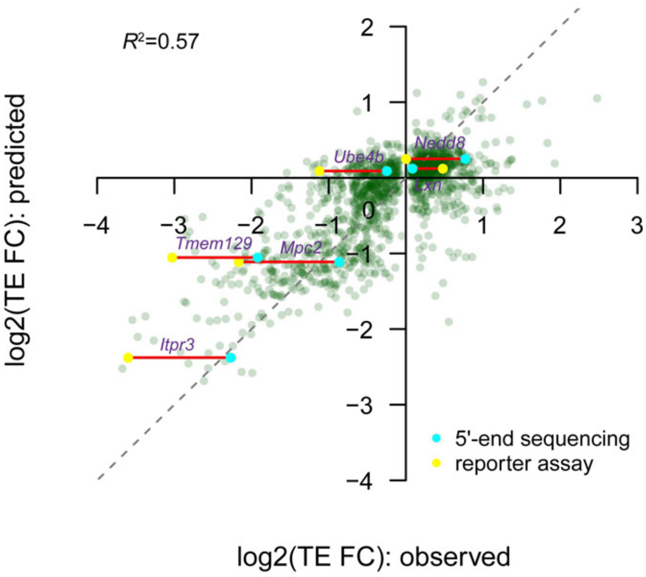

Figure EV7. Performance of the combinatory nonlinear regression model.

Same as Fig 6B, but in addition, we marked the six genes that were tested by luciferase reporter assay (Fig 2C) and containing unambiguously determined 5′UTR sequences (see Materials and Methods). The TE divergence values estimated based on 5′ end sequencing data are shown in cyan, and those based on reporter assay are shown in yellow.

© 2016 The Authors

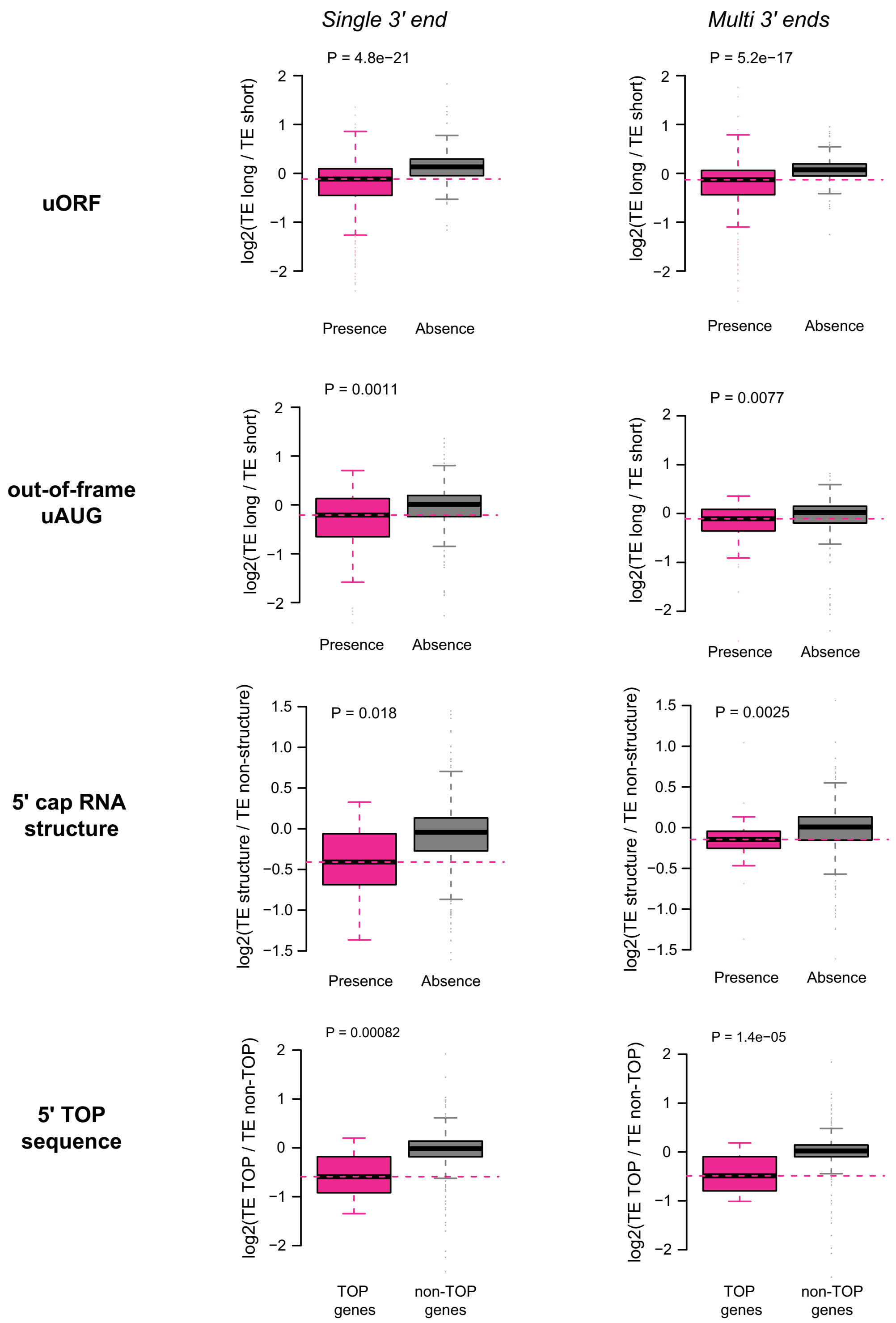

Figure EV8. Sequence features associated with translational regulation conferred significant impact in genes with single or multiple 3′ ends.
All the multi-TSS genes were separated into two groups based on whether only one or more 3′ end were identified in the study from Spies *et al* (2013). Similar to boxplots in Figs 4 and 5, but all comparisons were performed separately for the two groups (columns), one group contained the genes with only one 3′ end and the other group contained the genes with more than one 3′ end. Sequence features including uORF, out-of-frame uAUG, 5′ cap RNA structure, and 5′ TOP sequence (rows) were analyzed. Box edges represent quantiles, whiskers represent extreme data points no more than 1.5 times the interquartile range.

 © 2016 The Authors

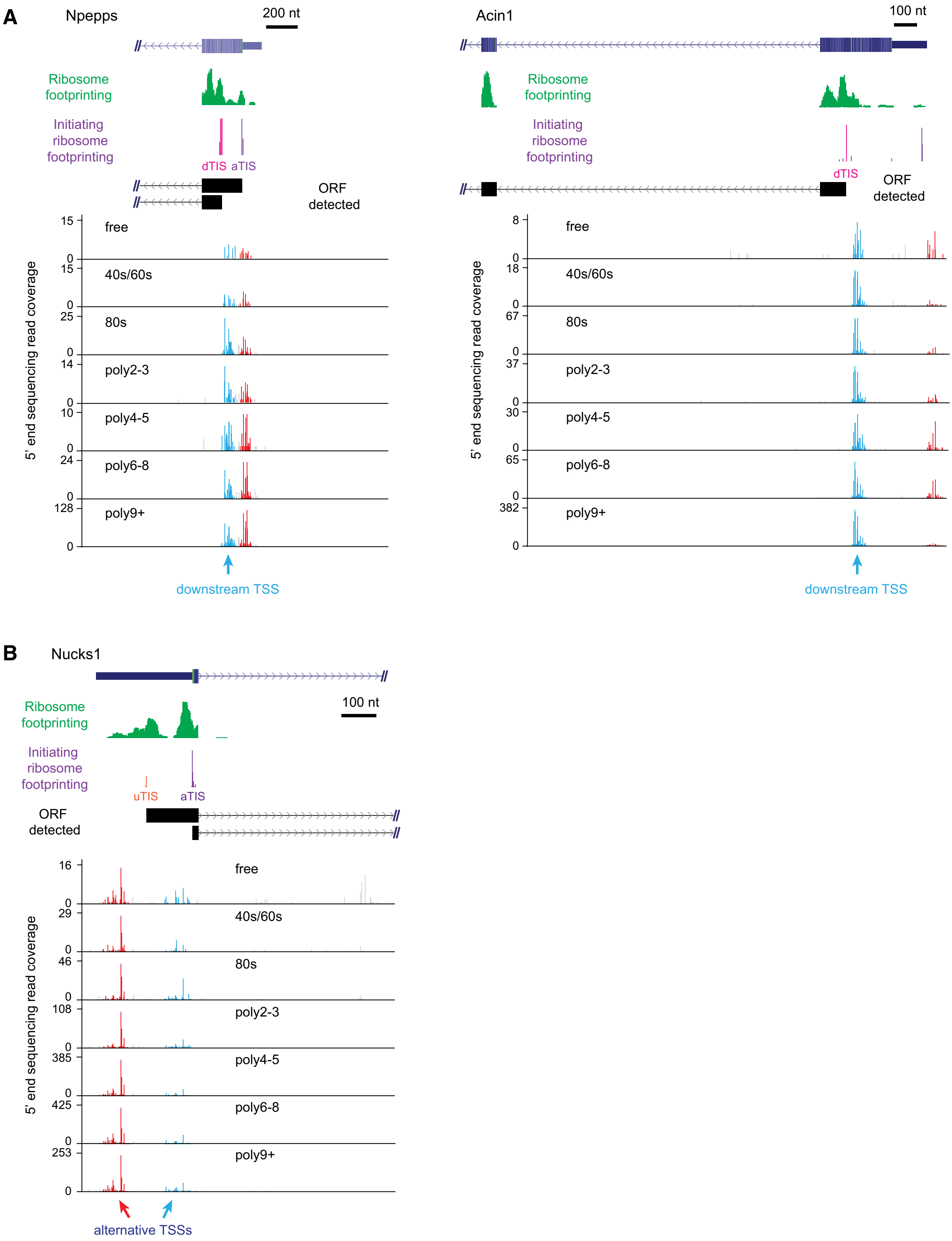

Figure EV9. Examples of alternative TSSs for protein N-terminal changes.
See also Table EV5.

A Downstream TSSs could lead to N-terminal truncated proteins. Two examples were shown here. The description of the two genes can be found in Table EV3.
B Alternative TSS could also lead to N-terminal extended proteins. One example was shown here. The description of this gene can be found in Table EV3.

© 2016 The Authors
