# Supplementary figures and images for: Pervasive isoform‐specific translational regulation via alternative transcription start sites in mammals
Source: Mol Syst Biol. 2016 Jul 18;12(7):875. doi: 10.15252/msb.20166941 (PMC4965872; doi:10.15252/msb.20166941)

# TOP\_Sequence

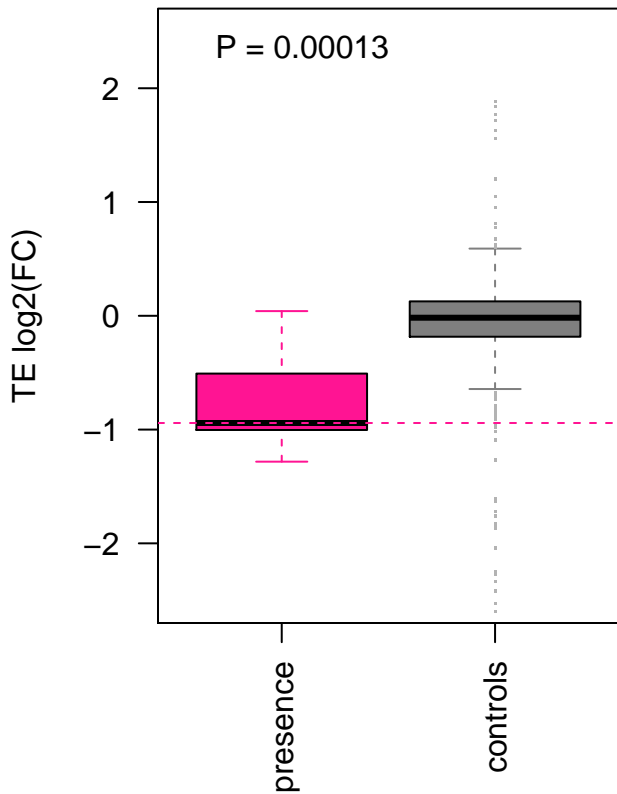

Supplement: Supplementary file 8 — Code EV1 [file MSB-12-875-s008.zip › GITHUB/Seq_feat/TOP.pdf]

# tot\_uORF

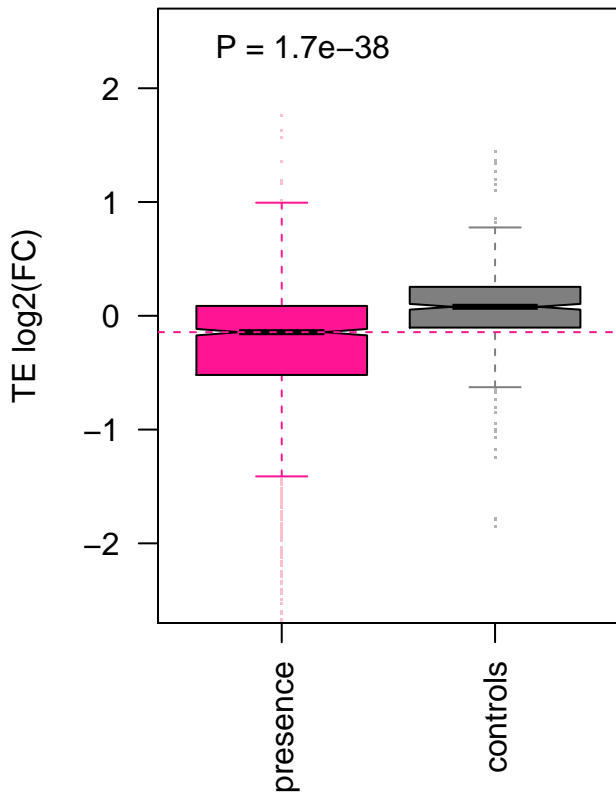

# inframe\_uAUG

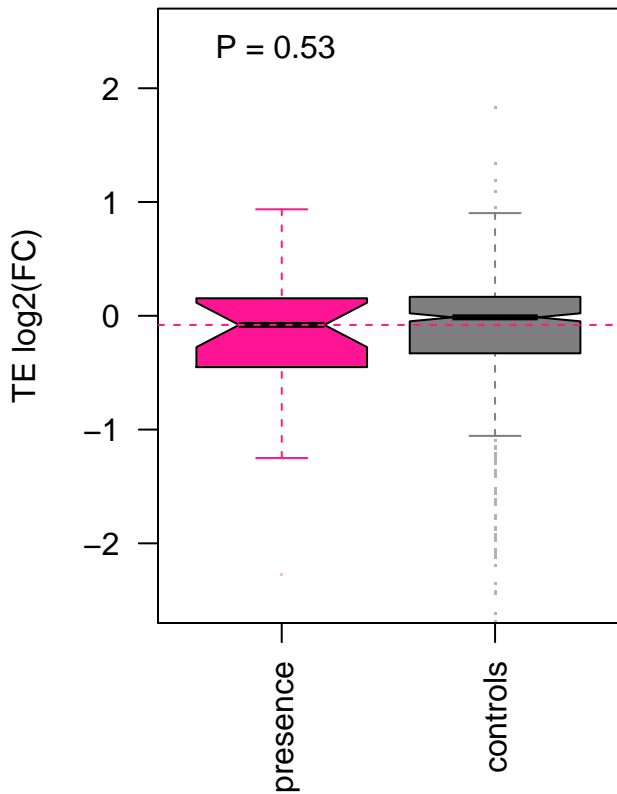

# outframe\_uAUG

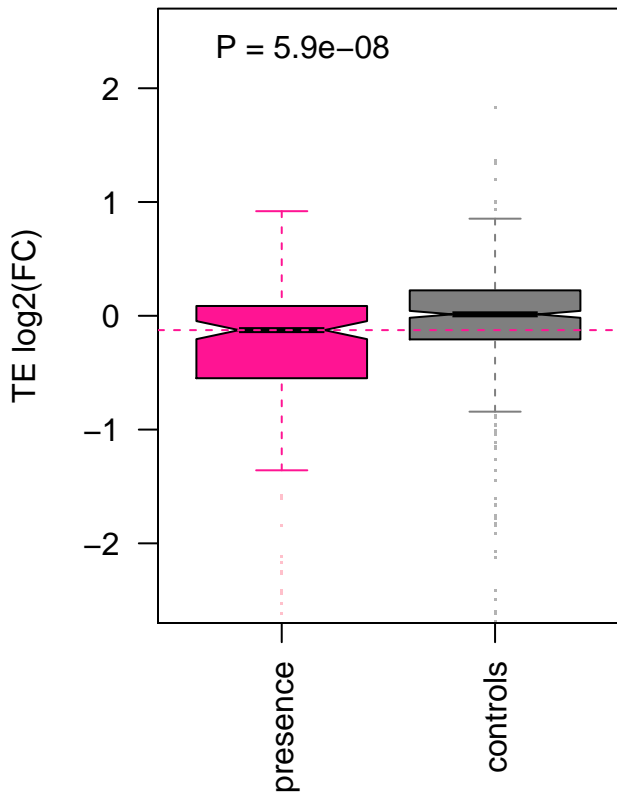

Supplement: Supplementary file 8 — Code EV1 [file MSB-12-875-s008.zip › GITHUB/Seq_feat/uORF.pdf]
